# MiR-4463 inhibits the migration of human aortic smooth muscle cells by AMOT

**DOI:** 10.1042/BSR20180150

**Published:** 2018-09-21

**Authors:** Xueqin Wang, Chao Du, Xuemei He, Xian Deng, Yanzheng He, Xiangyu Zhou

**Affiliations:** 1 Department of Vascular and Thyroid Surgery, The Affiliated Hospital of Southwest Medical University, Luzhou, Sichuan 646000, China; 2 Department of Basic Surgery, The People’s Hospital of Deyang City, Deyang, Sichuan 618000, China; 3 Collaborative Innovation Center for Prevention and Treatment of Cardiovascular Disease of Sichuan Province, Southwest Medical University, Luzhou, Sichuan 646000, China; 4 Key Laboratory of Medical Electrophysiology, Ministry of Education, Institute of Cardiovascular Research of Southwest Medical University, Luzhou, Sichuan 646000, China; 5 Medical Research Center, The Affiliated Hospital of Southwest Medical University, Luzhou, Sichuan 646000, China; 6 State Key Laboratory of Quality Research in Chinese Medicine, Faculty of Chinese Medicine, Macau University of Science and Technology, Avenida Wai Long, Taipa, Macau SAR 519020, P.R. China

**Keywords:** AMOT, human aortic smooth muscle cells, lower extremity atherosclerotic occlusive disease, miR-4463, migration

## Abstract

Aberrant vascular smooth muscle cell (VSMC) migration has been implicated in a variety of vascular disorders, while the signal pathways governing this process remain unclear. Here, we investigated whether miRNAs, which are strong post-transcriptional regulators of gene expression, could alter VSMC migration. We detected the expression of miR-4463 in the plasma of patients with atherosclerosis and in human aortic smooth muscle cells under hypoxia–ischemia condition, and investigated the migration effect and its downstream pathways. The results have shown that whether in clinical AS patients or hypoxic cells, the expression of miR-4463 was lower than that of normal group, then the number of migrating cells in the miR-4463 mimic intervention group was significantly decreased compared with the normal group and miR-4463 inhibitor instead. Furthermore, the expression of angiomotin (AMOT) in gastrocnemius muscle and femoral artery of patients was significantly higher than that of the control group. The protein level of AMOT in miR-4463 mimic intervention group was significantly decreased, and its level was reversed by inhibiting miR-4463. In summary, these results indicate that miR-4463 is a novel modulator of VSMC migration by targetting AMOT expression. Regulating miR-4463 or its specific downstream target genes in VSMCs may represent an attractive approach for the treatment of vascular diseases.

## Introduction

Atherosclerosis is a chronic inflammatory disease, characterized by atherosclerotic plaques that cause hardening and narrowing of the lumen, which is the overwhelming cause of morbidity and mortality in the elderly patients in developed and developing countries [1]. In the early stage of atherosclerosis, vascular smooth muscle cells (VSMCs) migrate from the middle membrane to the inner wall of the arterial wall, then abnormal proliferation and migration of VSMCs result in intimal thickening and narrowing at the end point [1,2]. At present, there are still many deficiencies in arteriosclerosis obliterans (ASO) treatment, regardless of clinical progress, such as regulating lipid metabolism, activating blood circulation, and intervene recanalization [3]. Therefore, more and more medical experts focussed on the development mechanism of arteriosclerosis, and they believed that inhibiting both the migration of VSMCs and the thickening of the intima from the molecular level may be benificial to prevent arteriosclerosis.

MiRNAs are endogenous non-coding small RNAs that can post-transcriptionally regulate entire sets of genes [3,4], which play an important role in cell proliferation, apoptosis, and migration. Many miRNAs have been confirmed in regulating the migration of VSMCs through different target genes or pathways. For example, miR-21 has been reported to promote proliferation and apoptosis of VSMCs by directly targetting programmed cell death 4 (PDCD4) [5]. Dong et al. [6] confirmed that the target gene Krüppel-like factor 4 (*KLF4*) of miR-146a was a basic regulator of the migration of VSMCs. Some recent studies also showed that podocalyxin-like protein (PODXL) is related to cell adhesion and migration. Zhu et al. [2] showed miR-24-3p analogs to significantly inhibit the platelet-derived growth factor-BB (PDGF-BB) induced VSMCs migration and proliferation through PODXL pathway. miR-221/222 can inhibit the proliferation and migration of VSMCs by inhibiting cyclin-dependent protein kinases (CDKs), p27, and p57 [4]. Researches on miRNA provide translational opportunities for innovative therapeutic and biomarker applications.

Currently, miRNAs are involved in various biological processes, which were regarded as diagnostic and prognostic biomarkers. In the vascular fields, our previous studies focussed on collecting plasma samples from patients with atherosclerosis to detect the expression of some interested miRNAs [6,7], and miR-4463 has begun to attract our attention. We found that miR-4463 has a significant change and it probably can be a potential biomarker for the early diagnosis of ASO. Even so, the exact regulating mechanism is obscure, and there is little report on whether miR-4463 is involved in the regulation of migration about VSMCs. Hence, the current study is to explore the underlying mechanisms between miR-4463 and migration in VSMCs based on our previous findings. After the successful construction of the cell model of hypoxia–ischemia to simulate the clinical ischemic disease, miR-4463 was down-regulated and up-regulated by transfecting miR-4463 inhibitor and mimic into cells. Then, cell Transwell, cell count, and flow cytometry were used to detect the functional status of the transfected cells, and some migration-related genes were investigated.

Through a series of experiments, we found that, compared with the control group miR-4463 expression in sclerosis patients plasma and in human aortic smooth muscle cells (HASMCs) under hypoxia–ischemia condition was lower, and migrating cells number in miR-4463 mimic group was significantly decreased compared with the control group. Based on the preliminary study on the migration downstream, we consider that the inhibitory effect of miR-4463 on migration of HASMCs may be due to up-regulation of angiomotin (AMOT) attracted and its downstream molecules.

## Materials and methods

### Cell culture and model making

In the present study, HASMCs (Sciencell, America) were selected as representative primary cells and cultured in smooth muscle cell medium (SMCM; Sciencell, America), which was supplemented with 2% heat-inactivated FBS, 1% cell growth factor, and 1% penicillin and streptomycin. We used trypsin (Gibco by Life Technologies, Australia) digestion passaged cell while adherent to approximately 80% and subcultured it, and cells at the 3–8 passages in the logarithmic phase were used for all experiments.

Atherosclerosis is a complex inflammatory disease, and the experimental method usually adopts hyperglycemia, angiotensin II, oxidized low density lipoprotein (ox-LDL), and hypoxia–ischemia to intervene and imitate [8–10]. To determine whether the expression of miR-4463 is important in ASO, we chose treated HASMCs with hypoxia–ischemia condition (5% O_2_ and 0% FBS) for 4 h to simulate the hypoxia condition *in vitro*.

The intracellular level of miR-4463 was up-regulated or down-regulated by transfecting miRNA mimic and inhibitor, respectively. Then, we chose two miRNAs of *Caenorhabditis elegans* as negative control (NC) according to the manufacturer’s protocol (Guangzhou RiboBio Co., Ltd., Guangzhou, China). Take the 24-hole plate for an example, cells were transfected with miR-4463 mimic/inhibitor or NC when the cell density reached 30–50% according to the instructions. First, miRNA storage liquid was mixed with 1× riboFECT™ CP Buffer and then the mixture was mixed with riboFECT™ CP Reagent. After incubation for 15 min at room temperature, the compound was added to the plate with moderate cell culture medium to make the concentration of miR-4463 mimic and inhibitor at 50 and 100 nmol/l, respectively. Transfection efficiency was determined by quantitative real-time PCR (RT-qPCR) 48 h later.

### RNA extraction and RT-qPCR analysis

The venous blood of 50 ASO patients and 50 normal persons were collected from the clinical sample. There was no statistical difference in sex and age between the two groups (Table 1). All the subjects received informed consent, and all the specimens were approved by the Ethics Committee of the Affiliated Hospital of the Southwest Medical University. The total RNA of tissues and HASMCs was extracted by TRIzol [9], and 500 ng RNA was used for reverse-transcription reaction (catalog number 218160; Qiagen GmbH, Hilden, Germany). Then, acquired cDNA was used for RT-qPCR according to the manufacturer’s protocol using the miScript SYBR Green PCR kit (catalog number 208054; Qiagen GmbH, Hilden, Germany). The primers of hsa-miR-4463 and U6 were purchased from Qiagen company (catalog number MIMAT0018987; MS00044996). RT-qPCR was performed in StepOnePlus version 2.2.3 Real-Time PCR system (Applied Biosystems; Thermo Fisher Scientific, Inc.) [11,12]. *C*_t_ value more than 35 was abandoned. Relative expression of target genes were obtained by normalization of the *C*_t_ values using the 2^−ΔΔ*C*_t_^ method, with *U6* as the reference gene [12].

**Table 1:** General data of study population

General information	ASO group (*n*=50)	Control group (*n*=50)
Age	73.72 ± 10.52	73.67 ± 7.92
Male/female (case)	27/23	26/24
Smoke (case)	10	8
Drink wine (case)	9	6
Hypertension (case)	17	0
Diabetes (case)	13	0

### Immunofluorescence

The adherent HASMCs were fixed with 4% paraformaldehyde and blocked with 10% BSA (Solarbio, China; catalog A8010). The cells were incubated overnight with the anti-smooth muscle antibody (SMA) (dilution 1:200) and incubated for 60 min with secondary antibody conjugated to the FITC fluorescent dye (dilution 1:400). DAPI (dilution 1:10) was used to stain the nucleus. Images were captured using an Olympus Fluorescence microscope (cellSens standard software version 1.6, DP80, Olympus Corporation, Tokyo, Japan) [13,14].

### Cell migration assays

In order to determine the effect of miR-4463 on HASMCs migration, transfected cells (3 × 10^5^) 200 µl were seeded into the upper chamber of a Transwell filter with 8-μm pores (Costar Corning, U.S.A.), the cells were deprived of serum for 24 h before being seeded. Then, SMCM containing 600 µl serum was added to the lower chamber [15,16]. Transwell chambers were incubated at 37°C for 24 h. After incubation, the cells that migrated through the filter pores were fixed in 4% paraformaldehyde and stained with 5% Crystal Violet. Non-migrating cells on the upper side of the filter were removed with cotton swabs. Migrating cells were imaged under an inverted phase-contrast microscope at 100× magnification (cellSens standard software version 1.6, DP72, Olympus Corporation, Tokyo, Japan).

### Western blot analysis

Total protein from HASMCs (2 × 10^6^ cell number) and tissue (100 mg) was extracted using the RIPA lysis buffer (Beyotime Institute of Biotechnology, China) [17,18]. The protein was separated by 12% SDS/PAGE and transferred on to PVDF membranes. Then incubated with primary antibodies, including anti-N-Cadherin, anti-E-Cadherin, anti-ERK, anti-P-ERK, anti-AKT, and anti-P-AKT (all for 1:1000; Cell Signaling Technology, China; cat: 13116, 3195S, 9102S, 9101S, 4691S, and 4060S), anti-AMOT (1:500; ABNOVA, China; cat: AADMOOAA6010700), anti-GAPDH for 1:2000 (Beyotime Institute of Biotechnology, China; cat: AF0006) overnight at 4°C. Then incubated with anti-rabbit IgG (Beyotime Institute of Biotechnology, China; cat: A0208) at 1:2000 dilution and anti-mouse IgG (Beyotime Institute of Biotechnology, China; cat: A0216) at 1:3000 dilution as secondary antibody for 1 h at 37°C. Finally, protein bands were visualized by Enhanced Chemiluminescence Detection Reagent (Bio-Rad Laboratories, Inc., Hercules, CA, U.S.A.), and blots were semi-quantitated by densitometric analysis using Quantity One version 4.6.2 software (Bio-Rad Laboratories, Inc.).

### Statistical analysis

Statistical analyses were performed using SPSS version 19.0 software (IBM Corp., Armonk, NY, U.S.A.). Data are presented as mean values ± S.D., and statistical significance for comparisons between two groups was calculated using the two-tailed *t* test. For multiple comparisons, ANOVA was performed. *P<0.05 was considered to be significant.

## Results

### The miR-4463 expression change and cell identification

**Figure 1:**
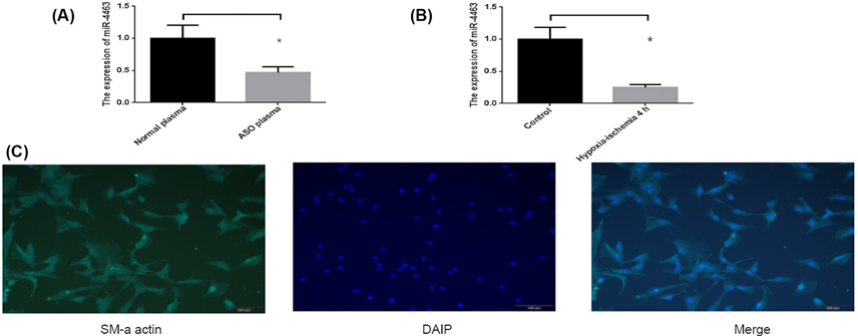
The miR-4463 expression was changed and cell identification (**A**) The expression level of miR-4463 in ASO patients’ than normal persons: decreased 53% (*P*=0.0145), *P<0.05. (**B**) The expression level of miR-4463 in HASMCs under hypoxia–ischemia condition than normal cells: declined 75% (*P*=0.0024), *P<0.05. (**C**) The HASMCs identification.

We examined the expression of miR-4463 in ASO patients and controls. The result showed that the expression level of miR-4463 was decreased by 53% (*P*=0.0145) in ASO patients than controls (Figure 1A). Then, HASMCs was selected to carry out the following experiments. The HASMCs were spindle or irregular triangles, the cytoplasms extend outward radially. Immunofluorescence staining of HASMCs with anti-SMA antibody which is an SMC marker, the results showed that strong positive expression and cell growth characteristics were normal distributions (Figure 1C). As for HASMCs, the miR-4463 was declined 75% (*P*=0.0024) treated with hypoxia–ischemia 4 h than untreated cells (Figure 1B). Our results show that miR-4463 was obviously changed whether in ASO patients or in HASMCs under hypoxia–ischemia condition compared with respective control, which suggest that miR-4463 positively participate in the pathogenesis of ASO.

### The miR-4463 inhibitor enhanced the HASMCs migration

**Figure 2:**
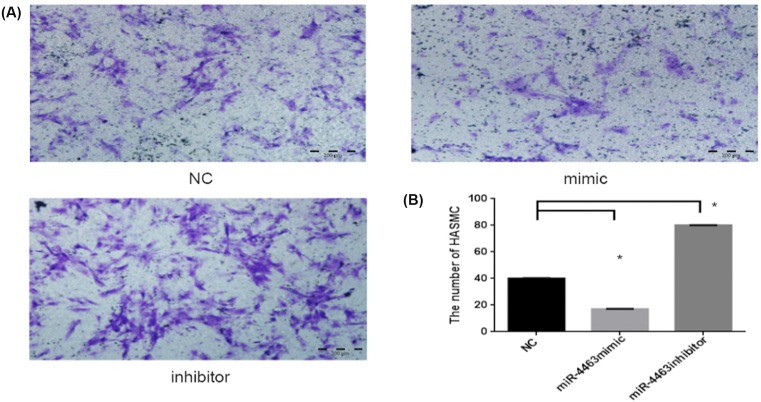
The HASMCs migration after transfection (**A**) Crystal Violet: HASMCs were pretreated with NC, miR-4463 mimic, and miR-4463 inhibitor, and under the phase-contrast microscope 100× after staining. Our result showed the cell number of miR-4463 mimic treated than NC group: decreased 57.5% (*P*<0.001), *P<0.05; but miR-4463 inhibitor group increased by 100.1% (*P*<0.001), “*P<0.05. (**B**) Corresponding histogram.

After transfection, the migrated cells through the Transwell filter pores were fixed and stained. At the same time, observing from the inverted phase-contrast microscope migrating cells images, our result showed that up-regulated miR-4463 declined HASMCs migration number, conversely, down-regulated miR-4463 promoted the migration effect. Through the statistical analysis of five fields vision of different batches, we found that the HASMCs migration number treated by miR-4463 mimic decreased 57.5% (*P*<0.001) than the NC group, but miR-4463 inhibitor group increased 100.1% (*P*<0.001) (Figure 2A is the Crystal Violet of HASMCs; (B) is the corresponding histogram of (A)). These results suggest that inhibiting miR-4463 plays an important role in enhancing HASMCs migration.

### MiR-4463 inhibitor promotes the expression of migration-related proteins

**Figure 3:**
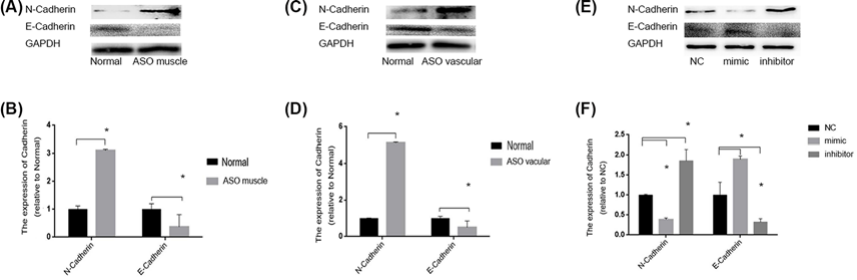
miR-4463 inhibitor regulates the expression of N-Cadherin and E-Cadherin (**A**) The expression of N-Cadherin in ASO patients’ gastrocnemius muscle tissue than normal persons up-regulated by 212.1% (*P*<0.001)), *P<0.05, and E-Cadherin declined by 64.0%(*P*=0.0031)), *P<0.05; (**C**) The expression of N-Cadherin in ASO patients’ femoral artery tissue than normal persons up-regulated by 411.7% (*P*<0.001)), *P<0.05, and E-Cadherin declined by 50.4% (*P*=0.0099)), *P<0.05; (**E**) The HASMCs after miR-4463 mimic transfected than NC: N-Cadherin down-regulated by 61.3% (*P*<0.001)), *P<0.05; E-Cadherin added 89.7% (*P*=0.0092)), *P<0.05; the miR-4463 inhibitor transfected than NC: N-Cadherin increased by 86.0% (*P*<0.001)), *P<0.05; E-Cadherin down-regulated by 67.3% (*P*=0.024)), *P<0.05. (**B**) Corresponding histogram of (A). (**D**) Corresponding histogram of (C). (**F**) Corresponding histogram of (**E**).

In order to further confirm the effect of miR-4463, first, the expression of N-Cadherin and E-Cadherin were detected, which are positively or negatively correlated with cell migration [19,20]. It is interesting that, no matter in gastrocnemius muscle tissue or in femoral arteries, the expression of N-Cadherin was up-regulated by 212.1 (*P*<0.001) and 411.7% (*P*<0.001) than normal tissues individually, E-Cadherin declined by 64.0 (*P*=0.0031) and 50.4% (*P*=0.0099), individually (Figure 3A,C) ((B) is the corresponding histogram of (A), and (D) is the corresponding histogram of (C)). In addition, consistent with tissues protein results, the expression of N-Cadherin in miR-4463 mimic-treated cells was down-regulated by 61.3% (*P*<0.001) than NC, then miR-4463 inhibitor increased by 86.0% (*P*<0.001). As for E-Cadherin, its expression in miR-4463 mimic-transfected cell was increased by 89.7% (*P*=0.0092) than NC and miR-4463 inhibitor decreased by 67.3% (*P*=0.024) (Figure 3E; (F) is the corresponding histogram of (E)).

**Figure 4:**
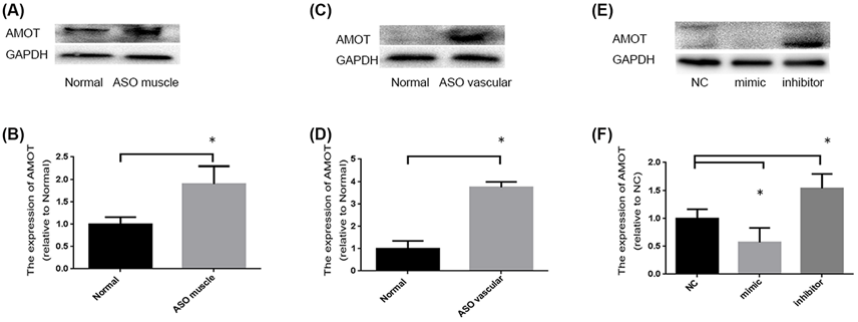
miR-4463 inhibitor promotes the expression of AMOT (**A**) The expression of E-Cadherin in ASO patients’ gastrocnemius muscle tissue than normal persons: increased 90.6% (*P*=0.02)), *P<0.05; (**C**) The expression of E-Cadherin in ASO patients’ femoral artery tissue than normal persons: 270.1% (*P*=0.0004)), *P<0.05; (**E**) The HASMCs after miR-4463 mimic transfected than NC: decreased 42.7% (*P*=0.007)), *P<0.05; the miR-4463 inhibitor transfected than NC: increased 54.2% (*P*=0.039)), *P<0.05. (**B**) Corresponding histogram of (A). (**D**) Corresponding histogram of (C). (**F**) Corresponding histogram of (E).

Next, we predicted the target genes of miR-4463 using TargetScan software (www.TargetScan.org), and AMOT attracted our attention for its biological relevance of migration. We then determined the expression of AMOT in the muscular and vascular tissues of patients and controls as well as in HASMCs. As shown in Figure 4, the AMOT protein was increased by 90.6% (*P*=0.02) and by 270.1% (*P*=0.0004) than normal tissue, respectively (Figure 4A,C) ((B) is the corresponding histogram of (A), and (D) is the corresponding histogram of (C)). Next, we also examined the proteins of N-Cadherin, E-Cadherin, and AMOT in transfected-HASMCs. And for HASMCs, miR-4463 mimic-transfected cells decreased 42.7% (*P*=0.007) than NC and miR-4463 inhibitor increased 54.2% (*P*=0.039) (Figure 4E; (F) is the corresponding histogram of (E)). Taken together, these results revealed that miR-4463 may inhibit HASMCs migration by down-regulating AMOT and its downstream effectors.

### Research on related pathways

**Figure 5:**
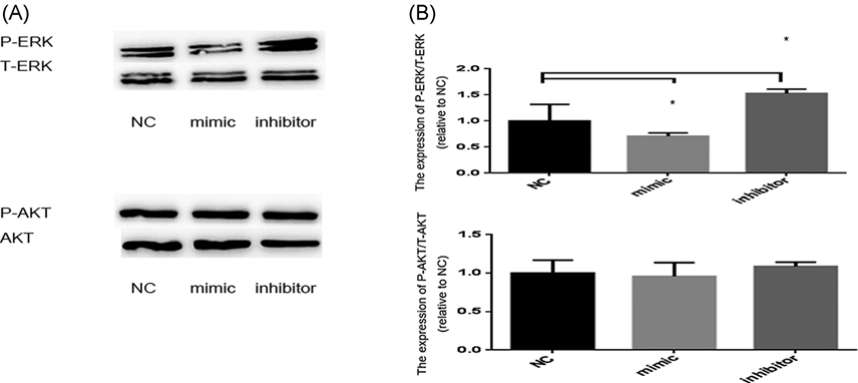
The effects of miR-4463 regulation on migration-related pathways (**A**) The expression level of P-ERK/ERK: miR-4463 mimic treated than NC: reduced 25.8% (*P*=0.02), *P<0.05; miR-4463 inhibitor treated than NC: raised 53.6% (*P*=0.0497)), *P<0.05; the expression level of P-AKT/AKT has no significant difference amongst NC, miR-4463 mimic, and miR-4463 inhibitor (mimic compared with NC, *P*=0.78; inhibitor compared with NC, *P*=0.44). (**B**) Corresponding histogram of (A).

Based on the study of AMOT-related migration downstream pathway, we speculated that the AMOT/AKT and AMOT/ERK may be the most likely targetted signal pathway. And the results showed that the expression level of P-AKT/AKT has no significant difference between NC, miR-4463 mimic, and miR-4463 inhibitor (mimic compared with NC, *P*=0.78; inhibitor compared with NC, *P*=0.44). However, the level of P-ERK/ERK in miR-4463 mimic group was reduced by 25.8% (*P*=0.02), while miR-4463 inhibitor group was raised by 53.6% than that of NC (*P*=0.0497) (Figure 5A is the expression level of P-ERK/ERK and P-AKT/AKT; (B) is the corresponding histogram of (A)). Above results suggest that miR-4463 may regulate VSMC migration, at least partly through the AMOT/ERK pathway.

## Discussion

The abnormal expression of miRNAs is involved in the regulation of proliferation, migration, and apoptosis of VSMCs. Our previous studies have found that the expression of miR-4463 was decreased in atherosclerosis serum than normal person serum [7]. Based on above results, we first examined the expression of miR-4463 in the tissues of ASO patients. As we expected, miR-4463 level in femoral artery and gastrocnemius muscle of atherosclerosis amputation patients was lower than that of accidental amputation patients. Then, HASMCs were induced in hypoxia–ischemia condition to simulate the hypoxic state of atherosclerosis-related diseases, and the expression of miR-4463 showed similar change with tissue samples. These results suggest that miR-4463 may be involved in the pathogenesis of atherosclerosis diseases.

The following study would like to explore the specific regulation mechanisms of miR-4463 from a molecular level. A series of cell functional experiments were performed to identify the miR-4463 implicated in the regulation of VSMC functions, including proliferation, apoptosis, and migration. In hypoxia–ischemia-induced HASMCs, there were no evidence that miR-4463 are associated with cell proliferation and apoptosis, but the Crystal Violet staining migration results showed that up-regulated miR-4463 inhibited the HASMCs migration, conversely, down-regulated miR-4463 promoted the migration. Numerous studies have shown that miRNAs are closely related to cell migration. Wang et al. [21] found that the miR-186-5p directly repressed FGF2 and RelA to inhibit the invasive and migratory abilities of GBM cells [22]. Li et al. [23] also suggested that the overexpression of miR-520c in N-87 and MKN-45 cancer cells significantly increased the proliferation, migration, and invasion ability of cancer cells *in vitro*. Combined with our results, we confirmed that miR-4463 inhibited the migration of HASMCs under hypoxia–ischemia condition.

It has been previously demonstrated that expression of the cell adhesion molecule, N-cadherin, may increase cell migration in VSMCs [19]. Many previous studies suggested that E-Cadherin is associated with maintaining cell morphology, cell motility, and adhesion, the effects are achieved by interacting with miRNAs [20]. Lyon et al. [19] have shown that inhibiting N-cadherin suppresses VSMC migration, at least in part by the induction of apoptosis both *in vitro* and *in vivo*. Zhang et al. [24] reported that miR-145 up-regulation could inhibit MG63 cells invasion and migration significantly through down-regulating the epithelial marker E-cadherin. Similar with above studies, our Western blot results also suggested the higher expression of N-Cadherin and lower expression of E-Cadherin whether in the lesion of femoral artery or in the gastrocnemius muscle in clinical atherosclerosis patients. Importantly, up-regulation of miR-4463 enhanced the expression of E-Cadherin and descended N-Cadherin, and miR-4463 inhibitor showed the opposite results in HASMCs. Therefore, the results further certified that miR-4463 inhibited the cell migration when the HASMCs suffered from hypoxia–ischemia, moreover, N-Cadherin and E-Cadherin were definitely involved in this process.

Based on TargetScan, *AMOT* is a gene that is related to angiogenesis, cell proliferation, and invasion, which is considered as a potential target gene regulated by miR-4463. The influence of AMOT on cell migration through YAP pathway has been confirmed by many literatures. The preliminary studies of Ruan et al. supported that miR-497 can act as a suppressor of *AMOT* gene expression in human osteosarcoma cells, resulting in suppression of tumor cell proliferation and invasion [25–27]. So, we hypothesized that miR-4463 might participate in HASMCs hypoxic condition by regulating AMOT. In order to test it, we first determined the protein level of AMOT in arterial and muscular tissues in ASO patients, and demonstrated that it was up-regulated. By both gain-of-function and loss-of-function approaches on HASMCs, we found that up-regulated miR-4463 suppressed the expression of AMOT, conversely, down-regulated miR-4463 reversed the effect. This further confirmed that miR-4463 may regulate VSMC migration through targetting AMOT directly or indirectly. To define the importance of AMOT in miR-4663-mediated VSMC function, we decided to further explore the downstream signal molecules of AMOT. Many basic experiments reported that AMOT plays an important role in angiogenesis and cell migration through Hippo-YAP pathway [26,28,29]. Especially, Zhang et al. [29] found that up-regulated AMOT can activate the ERK and AKT pathways by increased YAP expression in the development of colorectal cancer (CRC). Our results showed that there was no significant change in P-AKT/AKT after up/down-regulation of miR-4463. Interestingly, up-regulated miR-4463 inhibited the expression of P-ERK, and down-regulated miR-4463 restored it, which was in accordance with the above AMOT result. This suggests that miR-4463 may regulate VSMC migration, at least partly through the AMOT/ERK pathway.

Owing to some prior studies, significant novel insights are provided into the critical importance and functional complexities of miR-4463 in regulating VSMC migration as well as the development of vascular diseases. First, we found that the expression of miR-4463 was decreased in serum and oxygen-deprived condition, and the results were consistent with plasma and tissue levels. Then, overexpression of miR-4463 can inhibit the migration of VSMCs, and potential mechanism via inhibiting the target gene AMOT/ERK-related migration pathway. Our findings showed a novel insight into the regulatory effect of atherosclerosis-related migration, and the role of miR-4463 could be a potential therapeutic target for atherosclerosis.

## Abbreviations

AMOT: angiomotin
ASO: arteriosclerosis obliterans
HASMC: human aortic smooth muscle cell
NC: negative control
ox-LDL: oxidized low density lipoprotein
PODXL: podocalyxin-like protein
RT-qPCR: quantitative real-time PCR
SMA: anti-smooth muscle antibody
SMCM: smooth muscle cell medium
VSMC: vascular smooth muscle cell

## References

[1] Lee Y.T., Lin H.Y., Chan Y.W., Li K.H., To O.T., Yan B.P. (2017) Mouse models of atherosclerosis: a historical perspective and recent advances. Lipids Health Dis. 16, 12 10.1186/s12944-016-0402-5 28095860 PMC5240327

[2] Zhu X., Shan Z., Ma J., Wang M., Zhang C., Liu R. (2015) Investigating the role of the post-transcriptional gene regulator miR-24-3p in the proliferation, migration and apoptosis of human arterial smooth muscle cells in Arteriosclerosis obliterans. Cell. Physiol. Biochem. 36, 1359–1370 10.1159/000430302 26159387

[3] Chen K.C., Wang Y.S., Hu C.Y., Chang W.C., Liao Y.C., Dai C.Y. (2011) OxLDL up-regulates microRNA-29b, leading to epigenetic modifications of MMP-2/MMP-9 genes: a novel mechanism for cardiovascular diseases. FASEB J. 25, 1718–1728 10.1096/fj.10-174904 21266537

[4] Paola C., Devashish K., Hawthorne E.A., Liu S.L., Xu T., Rao S. (2013) miR-221/222 compensates for Skp2-mediated p27 degradation and is a primary target of cell cycle regulation by prostacyclin and cAMP. PLoS ONE 8, e56140 10.1371/journal.pone.0056140 23409140 PMC3567044

[5] Lin Y., Liu X., Cheng Y., Yang J., Huo Y. and Zhang C. (2009) Involvement of microRNAs in hydrogen peroxide-mediated gene regulation and cellular injury response in vascular smooth muscle cells. J. Biol. Chem. 284, 7903–7913 10.1074/jbc.M806920200 19158092 PMC2658083

[6] Dong S., Xiong W., Yuan J., Li J., Liu J. and Xu X. (2013) MiRNA-146a regulates the maturation and differentiation of vascular smooth muscle cells by targeting NF-kappaB expression. Mol. Med. Rep. 8, 407–412 10.3892/mmr.2013.1538 23784108

[7] He X.M., Zheng Y.Q., Liu S.Z., Liu Y., He Y.Z. and Zhou X.Y. (2015) Altered plasma microRNAs as novel biomarkers for Arteriosclerosis obliterans. J. Atheroscler. Thromb. 23, 196–206 26370316 10.5551/jat.30775

[8] Adesina S.E., Wade B.E., Bijli K.M., Kang B.Y., Williams C.R., Ma J. (2017) Hypoxia inhibits expression and function of mitochondrial thioredoxin 2 to promote pulmonary hypertension. Am. J. Physiol. Lung Cell. Mol. Physiol. 312, L599–L608 10.1152/ajplung.00258.2016 28130258 PMC5451594

[9] Kong S.S., Liu J.J., Yu X.J., Lu Y. and Zang W.J. (2012) Protection against ischemia-induced oxidative stress conferred by vagal stimulation in the rat heart: involvement of the AMPK-PKC pathway. Int. J. Mol. Sci. 13, 14311–14325 10.3390/ijms131114311 23203066 PMC3509582

[10] Zhang W.F., Zhu T.T., Xiong Y.W., Xiong A.Z., Ge X.Y., Hu C.P. (2017) Negative feedback regulation between microRNA let-7g and LOX-1 mediated hypoxia-induced PASMCs proliferation. Biochem. Biophys. Res. Commun. 488, 655–663 28108289 10.1016/j.bbrc.2017.01.073

[11] Arun A., Baumle V., Amelot G. and Nieberding C.M. (2015) Selection and validation of reference genes for qRT-PCR expression analysis of candidate genes involved in olfactory communication in the butterfly Bicyclus anynana. PLoS ONE 10, e0120401 10.1371/journal.pone.0120401 25793735 PMC4368739

[12] Wang Y. and Pan Y. (2014) Semi-supervised consensus clustering for gene expression data analysis. BioData Min 7, 7 10.1186/1756-0381-7-7 24920961 PMC4036113

[13] Shi T., Gao C., Yang L., Wei H., Jiao C., Fang F. (2017) Maresin 1 mitigates high glucose-induced mouse glomerular mesangial cell injury by inhibiting inflammation and fibrosis. Mediators Inflamm. 2017, 1–11 10.1155/2017/2438247 PMC5274668 28182085

[14] Abdollahi M., Ng T.S., Rezaeizadeh A., Aamidor S., Twigg S.M., Min D. (2017) Insulin treatment prevents wounding associated changes in tissue and circulating neutrophil MMP-9 and NGAL in diabetic rats. PLoS ONE 12, e0170951 10.1371/journal.pone.0170951 28182694 PMC5300126

[15] Seo H.H., Kim S.W., Lee C.Y., Lim K.H., Lee J., Choi E. (2017) A spleen tyrosine kinase inhibitor attenuates the proliferation and migration of vascular smooth muscle cells. Biol. Res. 50, 1 10.1186/s40659-016-0106-3 28100269 PMC5244549

[16] Liu W., Liu X., Luo M., Liu X., Luo Q., Tao H. (2017) dNK derived IFN-γ mediates VSMC migration and apoptosis via the induction of LncRNA MEG3: a role in uterovascular transformation. Placenta 50, 32 10.1016/j.placenta.2016.12.023 28161059

[17] Savoia C., Sada L. and Zezza L. (2011) Vascular inflammation and endothelial dysfunction in experimental hypertension. Int. J. Hypertens. 2011, 281240 21915370 10.4061/2011/281240 PMC3170891

[18] Fetahu I.S., Tennakoon S., Lines K.E., Groschel C., Aggarwal A., Mesteri I. (2016) miR-135b- and miR-146b-dependent silencing of calcium-sensing receptor expression in colorectal tumors. Int. J. Cancer 138, 137–145 10.1002/ijc.29681 26178670

[19] Lyon C.A., Koutsouki E., Aguilera C.M., Blaschuk O.W. and George S.J. (2010) Inhibition of N-cadherin retards smooth muscle cell migration and intimal thickening via induction of apoptosis. J. Vasc. Surg. 52, 1301–1309 10.1016/j.jvs.2010.05.096 20630685 PMC2977853

[20] Lyon C.A., Wadey K.S. and George S.J. (2015) Soluble N-cadherin: A novel inhibitor of VSMC proliferation and intimal thickening. Vasc. Pharmacol. 78, 53–62 10.1016/j.vph.2015.11.040 PMC4749540 26586312

[21] Wang F., Jiang H., Wang S. and Chen B., Dual functional microRNA-186-5p targets both FGF2 and RelA to suppress tumorigenesis of glioblastoma multiforme. Cell. Mol. Neurobiol. 2017, 1–10 10.1007/s10571-017-0474-4 PMC11482140 28213656

[22] Li X., Yao N., Zhang J. and Liu Z. (2015) MicroRNA-125b is involved in atherosclerosis obliterans *in vitro* by targeting podocalyxin. Mol. Med. Rep. 12, 561–568 10.3892/mmr.2015.3384 25738314

[23] Li Y.R., Wen L.Q., Wang Y., Zhou T.C., Ma N., Hou Z.H. (2016) MicroRNA-520c enhances cell proliferation, migration, and invasion by suppressing IRF2 in gastric cancer. FEBS Open Bio 6, 1257–1266 10.1002/2211-5463.12142 28203525 PMC5302056

[24] Zhang Z., Zhang M., Chen Q. and Zhang Q. (2017) Downregulation of microRNA-145 promotes epithelial-mesenchymal transition via regulating Snail in osteosarcoma. Cancer Gene Ther. 24, 83 28186090 10.1038/cgt.2017.1

[25] Ruan W.D., Wang P., Feng S., Xue Y. and Zhang B. (2016) MicroRNA-497 inhibits cell proliferation, migration, and invasion by targeting AMOT in human osteosarcoma cells. Onco Targets Ther. 9, 303–313 10.2147/OTT.S95204 26855583 PMC4727508

[26] Xiao J., Jin K., Wang J., Ma J., Zhang J., Jiang N. (2016) Conditional knockout of TFPI-1 in VSMCs of mice accelerates atherosclerosis by enhancing AMOT/YAP pathway. Int. J. Cardiol. 228, 605 10.1016/j.ijcard.2016.11.195 27875740

[27] Troyanovsky B., Levchenko T., Månsson G., Matvijenko O. and Holmgren L. (2001) Angiomotin: an angiostatin binding protein that regulates endothelial cell migration and tube formation. J. Cell Biol. 152, 1247–1254 10.1083/jcb.152.6.1247 11257124 PMC2199208

[28] Sasaki H. (2017) Roles and regulations of Hippo signaling during preimplantation mouse development. Dev. Growth Differ. 59, 12–20 10.1111/dgd.12335 28035666

[29] Zhang Y., Yuan J., Zhang X., Yan F., Huang M., Wang T. (2016) Angiomotin promotes the malignant potential of colon cancer cells by activating the YAP-ERK/PI3K-AKT signaling pathway. Oncol. Rep. 36, 3619–3626 10.3892/or.2016.5194 27779692

